# Sound field separation with cross measurement surfaces

**DOI:** 10.1371/journal.pone.0196837

**Published:** 2018-06-06

**Authors:** Jin Mao, Jinfu Du

**Affiliations:** School of Mechanical and Precision Instrument Engineering, Xi’an University of Technology, Xi’an, Shaanxi, China; Nanjing University, CHINA

## Abstract

With conventional near-field acoustical holography, it is impossible to identify sound pressure when the coherent sound sources are located on the same side of the array. This paper proposes a solution, using cross measurement surfaces to separate the sources based on the equivalent source method. Each equivalent source surface is built in the center of the corresponding original source with a spherical surface. According to the different transfer matrices between equivalent sources and points on holographic surfaces, the weighting of each equivalent source from coherent sources can be obtained. Numerical and experimental studies have been performed to test the method. For the sound pressure including noise after separation in the experiment, the calculation accuracy can be improved by reconstructing the pressure with Tikhonov regularization and the L-curve method. On the whole, a single source can be effectively separated from coherent sources using cross measurement.

## Introduction

In acoustics, near-field acoustical holography (NAH) is a widely used technique that reconstructs the acoustic field from data radiated by a source on the measurement surface [1, 2]. Sound field separation is one of the most useful methods in the NAH technique, which can separate the target source from coherent near-field sources [3].

Initially, a NAH separation method with double planes was developed to separate the pressure from the two sides of the array using spatial Fourier transform (SFT) [4–6]. Due to “wrap-around errors” caused by SFT in the calculation plane, statistically optimal NAH (SONAH) [7, 8] and equivalent source methods (ESM) [9–11] had been employed for the separation. Later, Jia [12, 13] separated the sound pressure through wave superposition with a single holographic surface, which was actually two surfaces: a holographic surface and a reconstruction surface calculated by holographic surface. However, it is impossible to separate the pressure radiated by sources on the same side of the array using the conventional separation method. The reason is that the ratio of transfer matrices of one source to the two measurement surfaces is the same as that of the other sources. If using planar hologram surfaces to separate the coherent sources on the same side of surfaces, the transfer function will be the same. Then, we would not separate the sound pressure radiated by target source successfully. Therefore, in order to separate the pressure from coherent sources that are located on the same side of the array, the cross measurement method with ESM is proposed.

A spherical surface with the same source center is set as the equivalent source surface. Through cross measurement, the transfer matrices of coherent sources based on ESM can be confirmed and the ratios of source matrices would be different. Thus, the weighting of each source could be calculated by a different sound pressure relationship. If a more accurate value of pressure is to be obtained, it can be recalculated with more data points on the reconstruction surface through Tikhonov regularization [14, 15] and the L-curve [16, 17].

## Theories

### Basic theory of ESM

The principle of ESM is simply expressed as follows: the acoustic field radiated by a complex radiator can be substituted by the acoustic superposition generated by a number of simple sources inside the radiator. The acoustic pressure can be represented using the Helmholtz formulation as follows:

$$p(r)=\int_{V}^{}j\rho ckq(r_{0})g(r,r_{0})dV(r_{0})$$ (1)

where

$$g(r,r_{0})=\frac{exp(-jk|r-r_{0}|)}{4\pi |r-r_{0}|}$$ (2)

is Green’s function between the simple source point *r*_0_ and the arbitrary acoustic field point *r*, $j=\sqrt{-1}$, *ρ* is the density of medium, *c* is the velocity of sound, *k* = 2*πf* / *c* is the wave number, *f* is the frequency of radiator, and *q*(*r*_0_) is the source strength.

Assuming that there are M microphones and N elementary sources, Eq (1) could be discretized as follows:

$$p(r_{m})=\sum_{n=1}^{N}j\rho ckq(r_{0n})\frac{exp(-jk|r_{m}-r_{0n}|)}{4\pi |r_{m}-r_{0n}|},\;m=1,\cdots ,M$$ (3)

Suppose that for the acoustic field reconstruction, the system of equations is underdetermined, i.e., *M* ≤ *N*. Eq (3) can then be written as a matrix:

$$P_{H}=GQ$$ (4)

where ***P***_*H*_ = [*p*(*r*_1_), ⋯, *p*(*r*_*m*_)]^*T*^, the superscript ^*T*^ indicates the transpose of a matrix, and ***Q*** = [*q*(*r*_01_), ⋯, *q*(*r*_0*n*_)]^*T*^. ***G*** is a transport matrix and is detailed as follows:

$$G=[\begin{array}{ccc} G(r_{1},r_{01}) & \cdots & G(r_{1},r_{0n})\\ \vdots & \ddots & \vdots \\ G(r_{m},r_{01}) & \cdots & G(r_{m},r_{0n}) \end{array}]$$ (5)

where

$$G(r_{m},r_{0n})=j\rho ck\frac{exp(-jk|r_{m}-r_{0n}|)}{4\pi |r_{m}-r_{0n}|}$$ (6)

The unknown source strength vector ***Q*** is obtained by inversion using the least norm solution [18] as follows:

$$Q=G^{H}{\left(GG^{H}+\lambda I\right)}^{-1}P_{H}$$ (7)

where ^*H*^ indicates the conjugate transpose of a matrix, *λ* is the regularization parameter, and ***I*** is the identity matrix. For *λ* ≠ 0, the equation is regarded as Tikhonov regularization, and the value of *λ* is determined by the L-curve.

Thus, the pressure on the reconstruction surface ***P***_*R*_ could be as follows:

$$P_{R}=G_{R}Q$$ (8)

where ***G***_*R*_ is the transfer matrix between the reconstruction surface data and equivalent source surface. The number of reconstruction surface data points could be more than the holographic surface with different grid spacing.

### Separation technique

To identify one source from two coherent sources located in the same direction relative to the microphones, two cross measurement layers are used. S1 Fig shows the measurement mode.

Similar to the conventional mode, where the measurement surface is perpendicular to the z-axis, pressures on the measurement surfaces of S1 Fig are related as shown in the equations below. *H*1 and *H*2 are holographic surfaces 1 and 2, respectively and *S*1 and *S*2 are source 1 and source 2, respectively.

$$P_{1}=P_{11}+P_{21}$$ (9)

$$P_{2}=P_{12}+P_{22}$$ (10)

Where ***P***_*j*_ indicates the pressure on surface *j* and ***P***_*ij*_ is the pressure on surface *j* radiated by single source *i*, where *i*, *j* = *1*,*2*. According to the principle of ESM, ***P***_*ij*_ could be expressed as follows:

$$P_{11}=G_{11}Q1$$ (11)

$$P_{21}=G_{21}Q2$$ (12)

$$P_{12}=G_{12}Q1$$ (13)

$$P_{22}=G_{22}Q2$$ (14)

where ***G***_*ij*_ represents the transpose matrix between equivalent sources and measurement points, and ***Q***1 and ***Q***2 are equivalent sources of single source *S*1 and single source *S*2, respectively.

Due to the relationship of the acoustic field radiated by sources *S*1 and *S*2, *P*_*ij*_ could be written in the relative form:

$$P_{12}=G_{12}G_{11}^{+}P_{11}$$ (15)

$$P_{21}=G_{21}G_{22}^{+}P_{22}$$ (16)

where

$$G_{11}^{+}={G_{11}^{H}(G_{11}G_{11}^{H}+\lambda I)}^{-1}$$ (17)

$$G_{22}^{+}={G_{22}^{H}(G_{22}G_{22}^{H}+\lambda I)}^{-1}$$ (18)

and where the regularization parameter *λ* is set to 0.001 [19].

Eqs (9) and (10) are expressed as follows:

$$P_{1}=P_{11}+G_{21}G_{22}^{+}P_{22}$$ (19)

$$P_{2}=G_{12}G_{11}^{+}P_{11}+P_{22}$$ (20)

In Eqs (19) and (20), ***P***_11_ is expressed as follows:

$$P_{11}=\frac{P_{1}-G_{21}G_{22}^{+}P_{2}}{I-G_{21}G_{22}^{+}G_{12}G_{11}^{+}}$$ (21)

Thus, ***P***_11_, the pressure on measurement surface *H*1 radiated by single source *S*1, is obtained. ***P***_22_ can also be obtained by the above method. Although the surface *H*1 is not parallel to the xy plane, a conventional surface could be built when reconstructing the acoustic field of a single source.

In particular, ***P***_11_ is the value errors including measurement error, random error and others. To get the more accurate pressure, it would need to be reconstructed in terms of Eq (8) using Tikhonov regularization and the L-curve.

## Numerical simulation

A numerical study using the MATLAB software was conducted to test the separation technique introduced in separation technique. On the basis of each material measurement mode as exhibited in S1 Fig, a holographic surface was generated consisting of a 6 × 6 discrete point grid with a 7 cm grid spacing. Pulsating balls *S*1 and *S*2 at 1000 Hz were located at (−0.15,0,0) *m* and (0.15,0,0) *m*, respectively. The radius and vibrating velocity were 0.01 m and 2.5 × 10^−2^ *m*/*s*. As the holographic surface *H*1 was not perpendicular to the z-axis, the bottom layer of the surface was located at *z*_*H*1*b*_ = 0.1 *m* and the top layer of the surface was located at *z*_*H*1*t*_ = 0.15 *m*. Similarly, the bottom and top layers of holographic surface *H*2 were located at *z*_*H*2*b*_ = 0.15 *m* and *z*_*H*2*t*_ = 0.1 *m*. Other bottom and top layers had been arranged at equal intervals, at a distance of 0.05 m on the z-axis. The SNR (signal to noise ratio) was set to 30 dB.

The reconstruction error with a relative average error level is defined in dB as

$$L_{err}=10∙{\text{log}}_{10}(\frac{\sum {\left|p_{i}^{true}-p_{i}\right|}^{2}}{\sum {\left|p_{i}^{true}\right|}^{2}})$$ (22)

where $p_{i}^{true}$ and *p*_*i*_ represent the theoretical pressure and calculation pressure after separation at the *i*th measurement point, respectively.

S2A Fig shows the three-dimensional diagram of pressure radiated by sources *S*1 and *S*2 on holographic surface *H*1, S2B Fig shows the theoretical pressure radiated by single source *S*1 on holographic surface *H*1, S2C Fig shows the calculation pressure after separation of *S*1 on holographic surface *H*1, and S2D Fig shows the xoz profile map of S2B and S2C Fig.

Comparing S2B and S2C Fig, the relative average level between theoretical value and calculation value is −31.29 dB. From S2D Fig, it can be seen that the calculation value is virtually identical to the theoretical value. Hence, by using the cross measurement separation technique with ESM, the sound pressure produced by a single source on a holographic surface could be separated out correctly from coherent sound sources.

Under the above numerical conditions, the simulations were performed several times over changing frequency, as shown in S3 Fig.

S3 Fig depicts the average error level on the holographic surface after separation relative to different frequencies (from 200 to 2000 Hz). In the middle range (from 600 to 1100 Hz), the separation results are more accurate than in other ranges. However, it is obvious that the errors, from the smallest -32.48 dB to the largest -28.25 dB, are consistently low in the whole range.

Assuming that the locations of *H*1’s bottom layer and *H*2’s top layer are the same and that the locations of *H*1’s top layer and *H*2’s bottom layer are the same, the location of *H*2’s bottom layer varies with the location of *H*1’s top layer. S4 Fig shows the average error level after separation relative to the changing location of -*H*1’s top layer with a fixed location (0.1 m) of -*H*1’s bottom layer.

S4 Fig shows low error when the location of *H*1’s bottom layer is stationary and the location of *H*1’s top layer changes from 0.11 m to 0.2 m. For simple and convenient measurement, *z*_*H*1*t*_ = 0.15 *m* is chosen.

## Practical measurement

Two loudspeakers with 1000 Hz were used as sources in the practical measurement. The holographic surface was set as a 6 × 6 element array with 0.1 m grid spacing. Set-up and location of all other instruments were the same as in the simulation, where *z*_*H*1*b*_ and *z*_*H*1*t*_ are 0.1 m and 0.15 m, respectively and *z*_*H*2*b*_ and *z*_*H*2*t*_ are 0.15 m and 0.1 m, respectively. S5 Fig shows the schematic diagram and photograph of the experiment.

The data on the two cross measurement surfaces were obtained by a fixed reference microphone and a group of three scanning microphones. The fixed microphone was employed to ensure that all the data measured by the scanning microphones had the correct phase during the whole process. The complex sound pressure on the measurement surfaces was then obtained after carrying out a FFT (Fast Fourier Transformation).

S6 Fig depicts the practical result. S6 Fig shows the pressure on the holographic surface *H*1, and it can be seen that there was noise during the measurement process. Thus, the pressure after separation included errors, as shown in S6B Fig. To obtain a more accurate result, Tikhonov regularization and the L-curve were used to reconstruct 169 data points with a 0.05 m grid spacing on the reconstruction surface, as shown in S6C Fig. Finally, the location of source *S*1 was recognized in S6D Fig, which shows the contour plot on the reconstruction surface.

## Conclusions

Conventional NAH separation methods make it impossible to obtain detailed sound pressure measurement of a single source among coherent sources that are located on the same side of the array. Based on ESM, the cross measurement method was proposed in this paper as a solution. Using the spherical surface set as equivalent source surface, we can build different equivalent source surfaces according to their location and size. Thus, the weighting of each equivalent source can be calculated as a function of pressure, where transfer matrices are determined by the location of equivalent sources and measurement points. Furthermore, the pressure radiated by a single source could be determined more accurately by reconstructing the calculation value with regularization. The effectiveness of this method has been examined by numerical simulation and experiment.

## Supporting information

S1 Fig Measurement mode. (TIF)

S2 Fig Pressure amplitude. (A) Data on Data on holographic surface *H*1. (B) Theoretical value radiated by source *S*1 on *H*1. (C) Calculated value after separation on *H*1. (D) Comparison section view on the xoz plane.
(TIF)

S3 Fig Calculation error with varying frequency. (TIF)

S4 Fig Calculation error with varying location of *H*1’s top layer. (TIF)

S5 Fig Experimental setup. (A) Schematic diagram. (B) Photograph.
(TIF)

S6 Fig Separation result. (A) Data on holographic surface. (B) Calculation value after separation on holographic surface. (C) Pressure on reconstruction surface. (D) Contour plot.
(TIF)
