# Supplementary figures and images for: Sound field separation with cross measurement surfaces
Source: PLoS One. 2018 Jun 6;13(6):e0196837. doi: 10.1371/journal.pone.0196837 (PMC5991362; doi:10.1371/journal.pone.0196837)

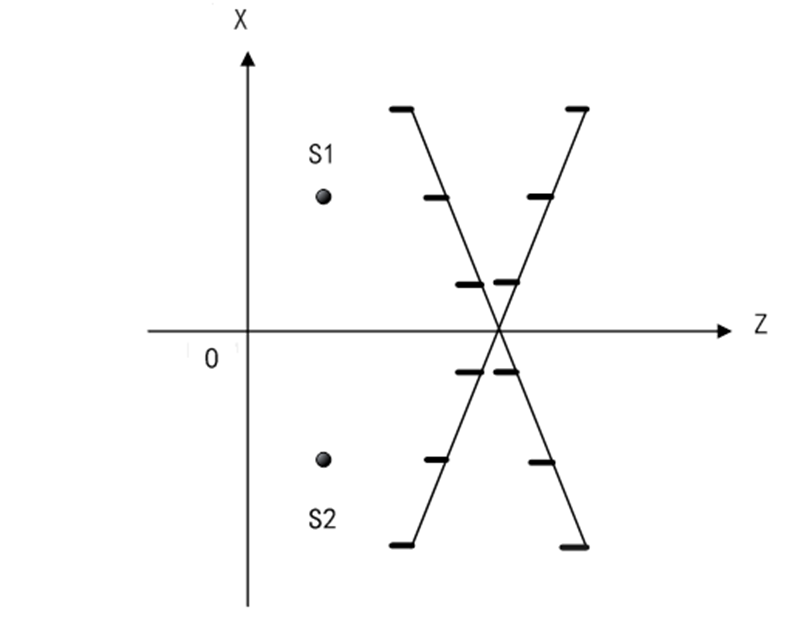

Supplement: S1 Fig — (TIF) [file pone.0196837.s001.tif]

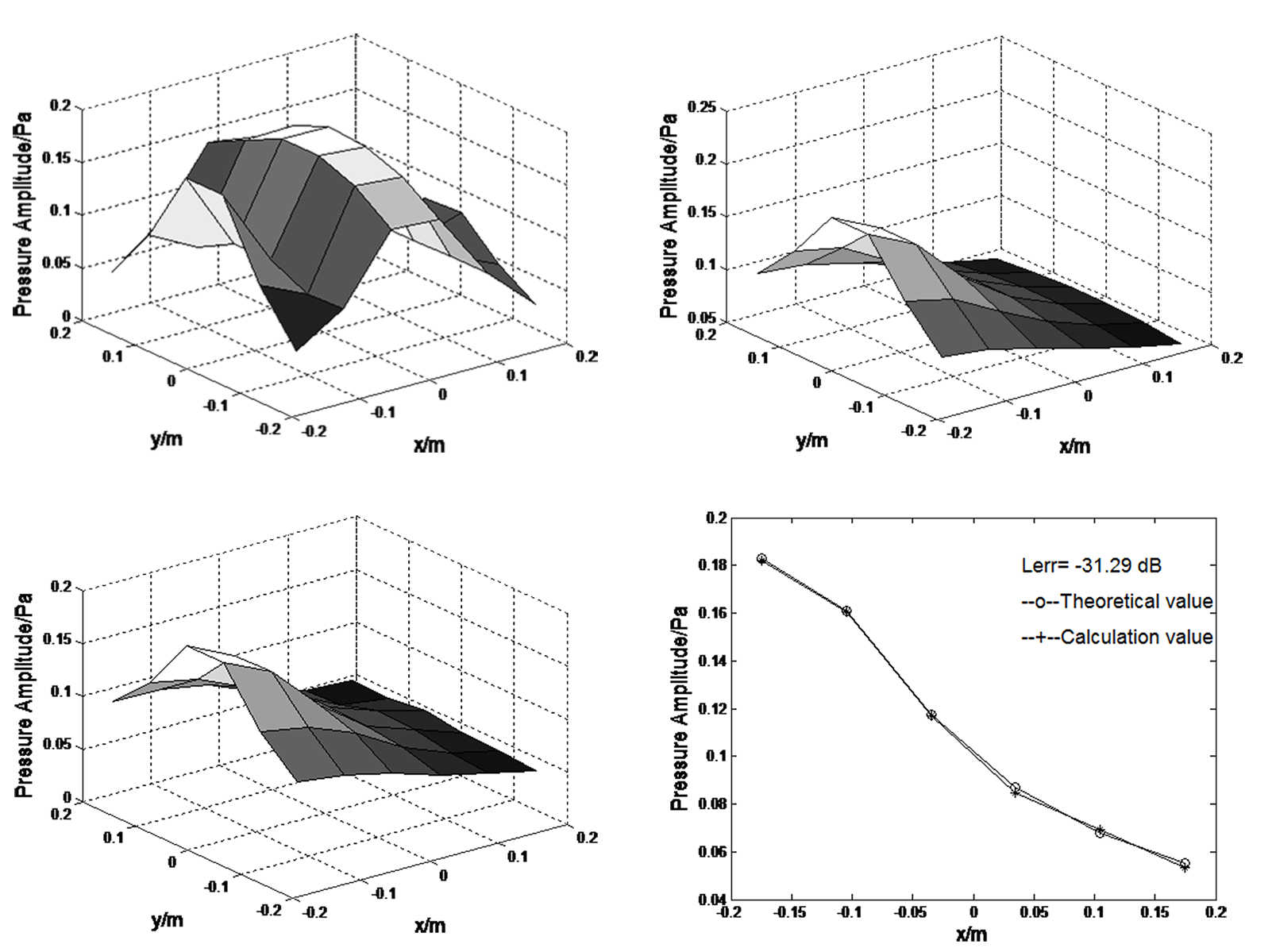

Supplement: S2 Fig — (A) Data on Data on holographic surface H1. (B) Theoretical value radiated by source S1 on H1. (C) Calculated value after separation on H1. (D) Comparison section view on the xoz plane. (TIF) [file pone.0196837.s002.tif]

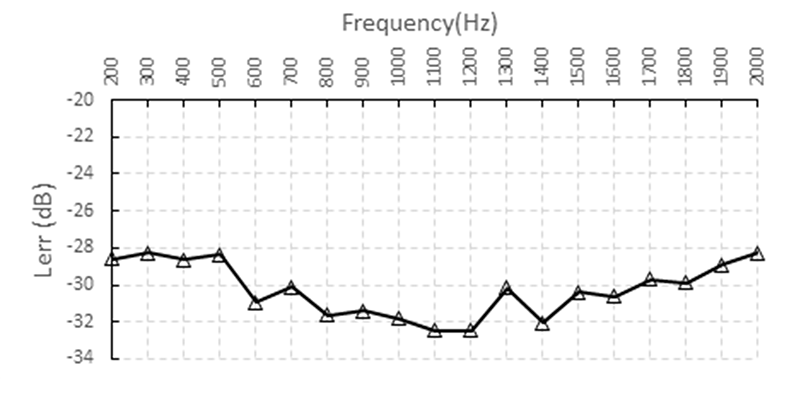

Supplement: S3 Fig — (TIF) [file pone.0196837.s003.tif]

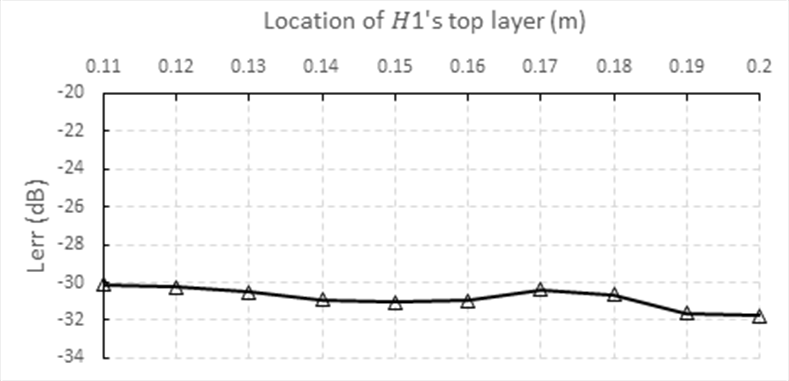

Supplement: S4 Fig — (TIF) [file pone.0196837.s004.tif]

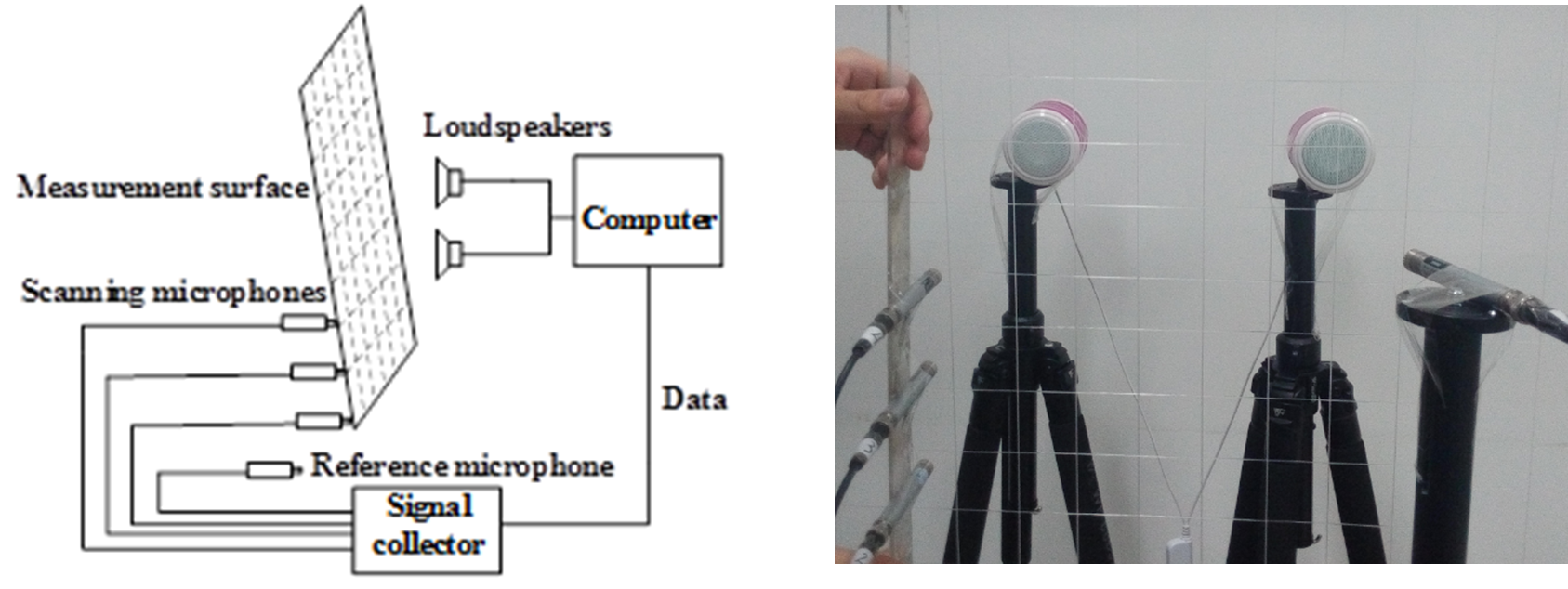

Supplement: S5 Fig — (A) Schematic diagram. (B) Photograph. (TIF) [file pone.0196837.s005.tif]

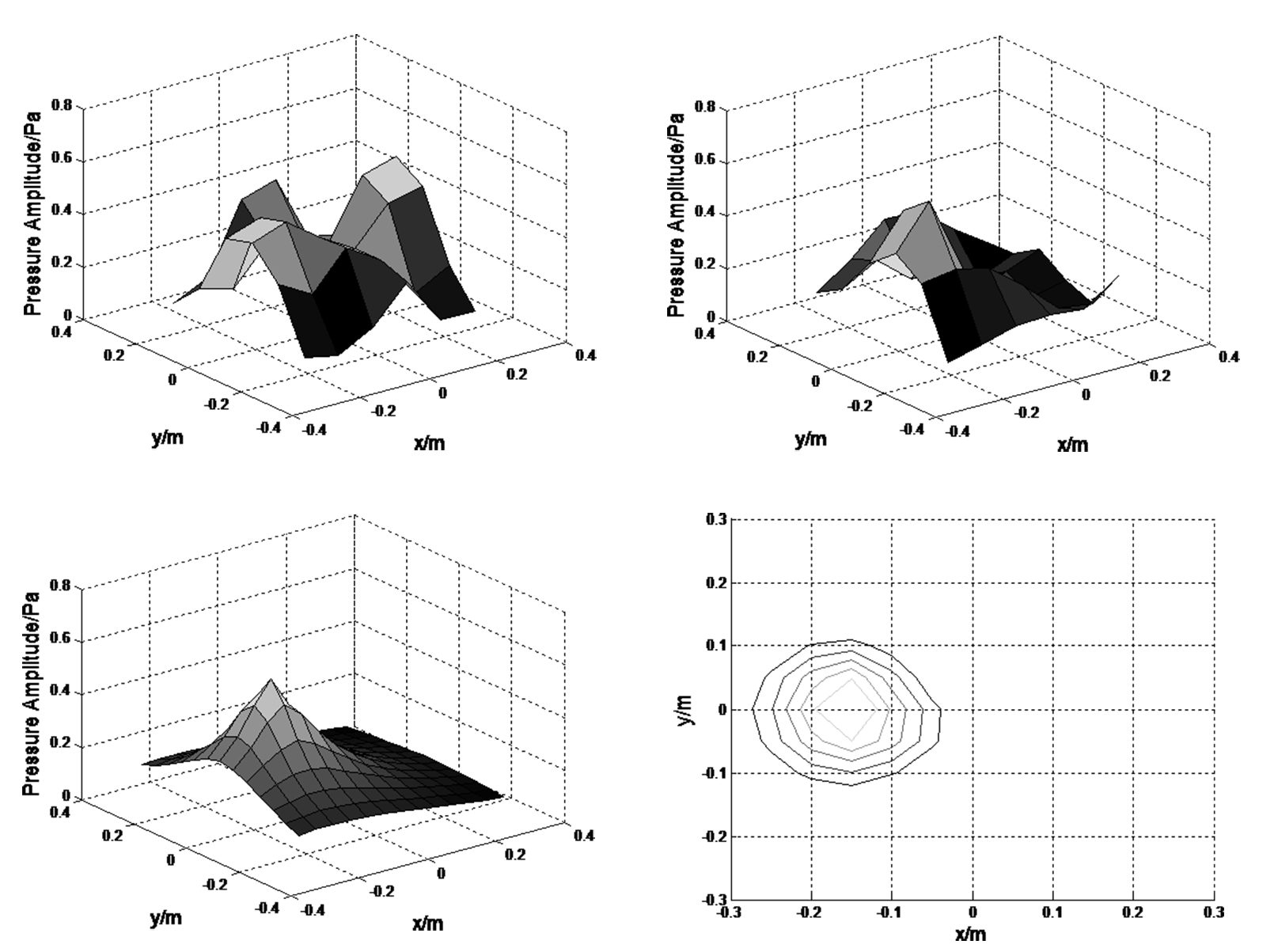

Supplement: S6 Fig — (A) Data on holographic surface. (B) Calculation value after separation on holographic surface. (C) Pressure on reconstruction surface. (D) Contour plot. (TIF) [file pone.0196837.s006.tif]
